# Prolonged Exposure to Particulate Pollution, Genes Associated with Glutathione Pathways, and DNA Methylation in a Cohort of Older Men

**DOI:** 10.1289/ehp.1002773

**Published:** 2011-03-08

**Authors:** Jaime Madrigano, Andrea Baccarelli, Murray A. Mittleman, Robert O. Wright, David Sparrow, Pantel S. Vokonas, Letizia Tarantini, Joel Schwartz

**Affiliations:** 1 Department of Epidemiology, and; 2 Department of Environmental Health, Harvard School of Public Health, Boston, Massachusetts, USA; 3 Cardiovascular Epidemiology Research Unit, Beth Israel Deaconess Medical Center, Boston, Massachusetts, USA; 4 Department of Pediatrics, Children’s Hospital Boston, Harvard Medical School, Boston, Massachusetts; USA; 5 Veterans Administration Normative Aging Study, VA Boston Healthcare System and Department of Medicine, Boston University School of Medicine, Boston, Massachusetts, USA; 6 Channing Laboratory, Brigham and Women’s Hospital, Harvard Medical School, Boston, Massachusetts, USA; 7 Center of Molecular and Genetic Epidemiology, Istituto Di Ricovero e Cura a Carattere Scientifico, Ca’ Granda Maggiore Policlinico Hospital Foundation and Department of Environmental and Occupational Health, University of Milan, Milan, Italy

**Keywords:** air pollution, DNA methylation, epigenetics, gene–environment. *Environ Health Perspect* 119:977–982 (2011). doi:10.1289/ehp.1002773 [Online 8 March 2011]

## Abstract

Background: DNA methylation is a potential pathway linking environmental exposures to disease. Exposure to particulate air pollution has been associated with increased cardiovascular morbidity and mortality, and lower blood DNA methylation has been found in processes related to cardiovascular morbidity.

Objective: We hypothesized that prolonged exposure to particulate pollution would be associated with hypomethylation of repetitive DNA elements and that this association would be modified by genes involved in glutathione metabolism and other host characteristics.

Methods: DNA methylation of the long interspersed nucleotide element–1 (LINE-1) and the short interspersed nucleotide element Alu were measured by quantitative polymerase chain reaction pyrosequencing in 1,406 blood samples from 706 elderly participants in the Normative Aging Study. We estimated changes in repetitive element DNA methylation associated with ambient particles (particulate matter ≤ 2.5 µm in aerodynamic diameter), black carbon (BC), and sulfates (SO_4_), with mixed models. We examined multiple exposure windows (1–6 months) before DNA methylation measurement. We investigated whether this association was modified by genotype and phenotype.

Results: An interquartile range (IQR) increase in BC over a 90-day period was associated with a decrease of 0.31% 5-methylcytosine (5mC) (95% confidence interval, 0.12–0.50%) in Alu. An IQR increase in SO_4_ over a 90-day period was associated with a decrease of 0.27% 5mC (0.02–0.52%) in LINE-1. The glutathione *S*-transferase mu-1–null genotype strengthened the association between BC and Alu hypomethylation.

Conclusion: Prolonged exposure to BC and SO_4_ particles was associated with hypomethylation of two types of repetitive elements.

The epigenome is increasingly being recognized as an important link between changes to the inherited genome and an evolving environment (Jirtle and Skinner 2007; Szyf 2007). Changes in patterns of DNA methylation have been well studied in cancer, and a global reduction of DNA methylation has been described as a striking feature of neoplasia (Wilson et al. 2007). Epigenetic modifications in other diseases are less understood but are increasingly being linked to processes related to cardiovascular disease (Castro et al. 2003), including atherosclerosis (Wierda et al. 2010) and changes in serum markers of endothelial function (Baccarelli et al. 2010a).

DNA methylation, the best-studied epigenetic process to date, occurs with the methylation of deoxycytosine bases to form deoxymethylcytosine. DNA methylation can be measured both in specific genes and in repetitive DNA sequences that are widespread throughout the genome (Bollati et al. 2007; Kazazian and Moran 1998). Repetitive elements, such as the long interspersed nucleotide element–1 (LINE-1) and Alu, a short interspersed nucleotide element, are retrotransposons, or sequences of DNA that can move around to different positions within the genome. They represent > 50% of the overall genome and are typically highly methylated, in order to suppress their expression (Schulz et al. 2006). LINE-1 elements are approximately 6,000 bp long and contain two transcribable open reading frames (ORFs), ORF1 (an RNA-binding protein) and ORF2 (an endonuclease/reverse transcriptase). Hypomethylation of LINE-1 promoters may activate expression of these proteins (Han and Boeke 2005). Demethylation of LINE-1 and Alu elements can increase their activity as transposable sequences, which may induce genomic alterations by insertion and/or homologous recombination and deregulate gene transcription when activated (Ostertag and Kazazian 2001).

In the present cohort, DNA hypomethylation has been associated with increased age (Bollati et al. 2009). In addition, DNA methylation can be influenced by dietary factors, pharmacologic agents, and environmental chemicals (Fornage 2007). Although evidence from *in vitro* and animal studies indicates that particulate matter (PM) and metals may affect global and gene-specific methylation (Belinsky et al. 2002; Chen et al. 2004), few human epidemiologic studies have examined the association between environmental pollutants and DNA methylation. Because epigenetic changes, once established, may be relatively stable, DNA methylation alterations induced by air pollution exposure may contribute to its long-term effects.

In this study, we sought to look at an exposure period longer than that examined in acute exposure studies but shorter than that used in studies of chronic exposure. We hypothesized that DNA hypomethylation would occur within 6 months of exposure to air pollution. We also wanted to determine if different types of PM pollutants would be associated with DNA hypomethylation over prolonged exposure periods compared with acute exposure periods. Hence, we looked at PM ≤ 2.5 µm in aerodynamic diameter (PM_2.5_) and two components: black carbon (BC) and sulfate (SO_4_).

Finally, genotype and phenotype have been shown to modify the effects of air pollution (Chahine et al. 2007; Schwartz et al. 2005). As a secondary aim, we hypothesized that genes involved in the glutathione metabolism pathway—glutathione *S*-transferase mu-1 (*GSTM1*), glutathione *S*-transferase theta-1 (*GSTT1*), and glutathione *S*-transferase pi-1 (*GSTP1*)—and other host characteristics would modify the association between prolonged exposure to PM air pollution and DNA methylation.

## Materials and Methods

*Study population.* This study included 706 elderly men who, as of March 1999, were active participants in the Normative Aging Study (NAS). The NAS cohort was established by the Veterans’ Administration in 1961, which enrolled men 21–80 years of age from the greater Boston, Massachusetts, area who were free of known chronic medical conditions (Bell et al. 1972). Since the time of enrollment, participants have had comprehensive clinical examinations at 3- to 5-year intervals. Further details can be found elsewhere (Park et al. 2005). By 1999, when measurements of DNA methylation began, 668 original participants had died and a number of subjects were no longer being followed, primarily because they moved out of the region after retirement. In examinations that took place between March 1999 and November 2007, 782 active participants agreed to donate at least one blood sample that was used for DNA methylation analysis. After excluding men with missing information for any of the covariates of interest, the final analysis included 706 individuals with 1,406 observations. Of these individuals, 200 (28%) had three observations, 300 (42%) had two observations, and 206 (30%) had only one observation. NAS participants reported to the study center on the morning of their scheduled examinations. A physician elicited a complete medical history, and smoking history was assessed using the American Thoracic Society questionnaire (Ferris 1978). All participants had given written informed consent. This study was reviewed and approved by the institutional review boards of all participating institutions.

*Air pollution measurements.* Air pollutants measured were ambient PM_2.5_, BC, and SO_4_. Measurements were obtained from a stationary monitoring source located at the Harvard School of Public Health (HSPH), < 1 km from the examination site where the subject visits took place. The median distance of the participant homes from the central site monitoring station was 20.5 km, with an interquartile range (IQR) of 10.5–37.9 km.

PM_2.5_ was measured continuously using a Tapered Element Oscillating Microbalance (model 1400A; Rupprecht and Patashnick Co., East Greenbush, NY, USA), operated at 50°C with two 4-L/min PM_2.5_ impactors before the inlet. A season-specific correction, based on collocated gravimetric samplers, was used to correct for loss of semivolatile particles in the monitor. BC was measured continuously using an aethalometer (AE-14; Magee Scientific Inc., Berkeley, CA, USA). Hourly averages were calculated based on the continuous measurements. Daily averages were based on a mean of the hourly averages in the 24-hr period before the examination. For PM_2.5_ and BC, the 24-hr period was always calculated from 0800 to 0800 hours. Because fasting blood samples are drawn during examinations, all visits are scheduled for the morning, so this period is very close to the visit time.

Measurements for BC were available from 1 January 1995 through November 2007, with a gap from March 1997 to December 1999, when the pollution monitor was shut down. No data are available for that time period. During the months of April through December 1999 the Massachusetts State Department of Environment operated an identical monitor at a nearby site (~ 3 km apart) in Roxbury, Massachusetts. We used monitoring data from that site for this period, after calibrating the measurements to the HSPH monitor using a linear regression of data from the time period when both monitors were operating. For the remaining period when missing data occurred for either BC or PM_2.5_ (~ 10% of hourly measurements), levels were imputed through a linear regression model (Zanobetti and Schwartz 2006).

SO_4_ data were available from 25 September 1999 through the end of the study period, November 2007. From 25 September 1999 to 2 February 2004, particulate SO_4_ was measured using the Harvard/EPA Denuder System (HEADS), which samples inorganic gaseous and particulate species in air. Concentrations of SO_4_ were calculated from the net SO_4_ ion concentration on the Teflon filter and the net volume of ambient air sampled. Samples were collected for 24-hr periods (always calculated from 0900 to 0900 hours). From 1 January 2003 through November 2007, daily particulate filter samples were analyzed by X-ray fluorescence (XRF) spectroscopy for elemental components. From these samples, we multiplied the mass of sulfur by 3 to obtain the mass of SO_4_. For days when both HEADS impactors and XRF were in operation, we used linear regression and determined that the measurements had a 1-to-1 slope and *R*^2^ > 0.9, indicating a high correlation between the two monitoring methods. Hence, XRF measurements were used during this period of overlap. Air temperature and dew point temperature for each day were obtained from the National Climatic Data Center (http://www.ncdc.noaa.gov/oa/ncdc.html).

*Laboratory methods.* DNA was extracted from stored frozen buffy coat of 7 mL whole blood, using QiAmp DNA blood kits (Qiagen, Hilden, Germany). DNA methylation was quantitated using bisulfite-polymerase chain reaction (PCR) and pyrosequencing, using previously described primers and conditions (Bollati et al. 2007; Yang et al. 2004). PCR primers were designed toward consensus LINE-1 and Alu sequences and allowed for the amplification of a representative pool of repetitive elements to serve as a surrogate for diffuse genomic DNA methylation changes. The degree of methylation was expressed as 5-methylated cytosines (5mC) as a percentage of the sum of methylated and unmethylated cytosines. This method quantitatively assessed the proportion of methylated sites in LINE-1 and Alu repetitive elements dispersed throughout the genome. Non-CpG cytosine residues were used as built-in controls to verify bisulfite conversion. Compared with other common methods of DNA methylation analysis, pyrosequencing-based assays have the advantage of producing individual measures of methylation at more than one CpG dinucleotide, thus more accurately reflecting DNA methylation in the region. For both Alu and LINE-1, we measured the percentage of 5mC at each of three CpG dinucleotide positions that are repeated over the human genome with the sequence of interest. Each sample was tested in two replicates, and their average was used in the statistical analysis.

*Genotyping methods. GSTM1* and *GSTT1* were analyzed as deletion versus no deletion. Two single-nucleotide polymorphisms on *GSTP1* were evaluated. These were analyzed as any variant versus none. For further details on genotyping, see Supplemental Material (doi:10.1289/ehp.1002773).

*Other covariates.* Questionnaires evaluated smoking habits and medication use, with responses confirmed by an on-site physician. Covariate information, specifically age, body mass index (BMI), cigarette smoking, medication use, and alcohol intake, were assessed at each medical examination.

*Statistical analysis.* To determine whether prolonged pollutant exposure was associated with changes in methylation, we examined multiple moving-averaged exposures of PM_2.5_, BC, and SO_4_. The moving average is the mean exposure for the time period before each examination. Separate models were created for each pollutant and for each of the following moving-average exposure windows: 28, 45, 60, 90, and 180 days preceding the clinical examination. Because of repeated measures of Alu and LINE-1 methylation for each participant and separate measurements at three CpG dinucleotide positions, our data may lack independence. Accordingly, we fit mixed-effects models with two random intercepts, to capture the correlation among measurements within the same subject, or same location within promoter region. We assumed the following:

*Y_ijk_* = β_0_ + *µ_k_* + *µ_i_* + β_1_ pollutant   + β_2_*X*_2_+ . . . + β*_p_X_p_* + ε*_ijk_*, [1]

where *Y_ijk_* is the measured value of Alu or LINE-1 at CpG dinucleotide position *i* at visit *j* of subject *k*; β_0_ is the overall intercept; *µ_k_* is the separate random intercept for subject that captures the correlation among measurements within the same subject; *µ_i_* is the separate random intercept for each CpG dinucleotide position, which captures the correlation among measurements at the same dinucleotide position; and *X*_2_ – *X_p_* are the covariates. We note that random effects from a mixed model could be biased when some participants have only one measurement. However, here we were interested only in the valid fixed effects.

The following potential confounders or predictors of Alu and LINE-1 methylation were chosen *a priori* based on previous literature (Baccarelli et al. 2009; Bollati et al. 2009; Zhu et al. 2010) and included in the analysis: season (with indicator variables); linear and quadratic terms for apparent temperature; a composite index of human discomfort due to combined heat and high humidity, calculated from air and dew point temperature (Zanobetti and Schwartz 2008); a linear term for time to capture long-term trends; age (as a continuous variable); smoking (indicators for current and former smoking and a continuous variable for pack-years); BMI (as a continuous variable); prescription medication (indicators for patient taking statin medication, medication for hypertension, or diabetes); alcohol intake (> 2 drinks per day); an indicator for laboratory batch; and percent lymphocytes and percent neutrophils (the latter were included to control variation among leukocyte populations in LINE-1 and Alu methylation). Individual covariates such as age were all time varying; hence, age and time trend together capture both changes in methylation with age and secular trends in methylation. All mixed-effects models were conducted using the PROC MIXED procedure in SAS (version 9.2; SAS Institute Inc., Cary, NC, USA).

We examined the modifying effects of variants in genes related to glutathione metabolism. Effect measure modification was examined by a cross-product term of any variant versus no variant (or deletion vs. no deletion) and a linear term for each air pollutant. We also examined the modifying effects of host characteristics [current smoking, obesity (BMI ≥ 30), and hypertensive medication] through a cross-product term of an indicator of having the characteristic and a linear term for each air pollutant. For this analysis, we chose the air pollutant exposure periods where we saw most of the significant main effects, that is, an association with statistical significance of *p* ≤ 0.05. Separate models were fitted for each combination of air pollutant and genetic variant or host characteristic. We examined interaction between BC and SO_4_, separately, during the periods 45, 60, and 90 days before exposure, and each genetic variant and defined effect modification as present if the *p*-value for the cross-product term was ≤ 0.05.

*Sensitivity analysis.* Finally, we performed two sensitivity analyses. In the first, we restricted our data to participants (*n* = 501) who live within 40 km of the primary stationary air pollution monitor. In the second, we adjusted for season through the use of a sine and cosine function of time in our models.

## Results

Table 1 lists baseline characteristics of the study population. The mean age of participants was 72 years. The mean levels of LINE-1 and Alu methylation were 77.5% and 26.1% 5mC, respectively. The methylation of Alu and LINE-1 was negatively correlated (ρ = –0.29). Means and IQRs of pollutants varied by season and are provided in the Supplemental Material [Table S.1 (doi:10.1289/ehp.1002773)]. Table 2 shows IQRs used in the calculation of our effect estimates for each time window, along with our results. The IQRs in our study are lower than the 10-µg/m^3^ exposure increment used in many studies examining PM_2.5_ and morbidity and mortality (Brook et al. 2010), and the levels of PM_2.5_ in the Boston metropolitan area are generally lower than the U.S. average and well within regulatory standards.

**Table 1:** Baseline characteristics of subjects in the NAS [*n* = 706; mean ± SD or *n* (%)].

Table 1. Baseline characteristics of subjects in the NAS [*n* = 706; mean ± SD or *n* (%)].	
Characteristic	Baseline visit
Age (years)	72.2 ± 6.8
BMI (kg/m^2^)	28.3 ± 4.1
Obesity	191 (27.1)
Smoking status	
Never	201 (28.5)
Former	475 (67.3)
Current	30 (4.2)
Cumulative smoking (pack-years)*a*	30.0 ± 27.4
Alcohol consumption	
< 2 drinks per day	577 (81.7)
≥ 2 drinks per day	129 (18.3)
Taking diabetes medication	60 (8.5)
Taking hypertensive medication*b*	411 (58.2)
Taking statin medication	247 (35.0)
DNA methylation (% 5mC)	
LINE-1	77.5 ± 2.5
Alu	26.1 ± 1.1
*GSTM1* deletion	368 (52.1)
*GSTP1 *	
rs1695, any variant	347 (49.2)
rs1799811, any variant	90 (12.7)
*GSTT1* deletion	130 (18.4)
**a**Among current or former smokers. **b**Includes angiotensin-converting enzyme inhibitors, β-adrenergic blocking agents, calcium channel–blocking agents, and diuretics.	

**Table 2:** Change in LINE-1 and Alu methylation (%5mC) associated with an IQR increase in pollutant.

Table 2. Change in LINE-1 and Alu methylation (%5mC) associated with an IQR increase in pollutant.						
		β-Coefficient*a* (95% CI)				
Exposure (average)		LINE-1		Alu		IQR (µg/m^3^)
PM_2.5_						
28 days		–0.01 (–0.16 to 0.13)		0.00 (–0.10 to 0.09)		3.33
45 days		–0.04 (–0.18 to 0.11)		0.02 (–0.08 to 0.11)		2.97
60 days		–0.05 (–0.20 to 0.10)		0.03 (–0.07 to 0.13)		2.66
90 days		0.03 (–0.12 to 0.18)		0.03 (–0.07 to 0.13)		2.32
180 days		0.07 (–0.05 to 0.19)		0.07 (–0.01 to 0.15)		1.53
BC						
28 days		–0.22 (–0.43 to –0.01)*		–0.03 (–0.17 to 0.10)		0.27
45 days		–0.31 (–0.57 to –0.05)*		–0.17 (–0.34 to 0.00)*		0.27
60 days		–0.29 (–0.56 to –0.02)*		–0.21 (–0.39 to –0.03)*		0.25
90 days		–0.21 (–0.50 to 0.09)		–0.31 (–0.50 to –0.12)*		0.24
180 days		0.20 (–0.16 to 0.55)		–0.10 (–0.33 to 0.13)		0.21
SO_4_						
28 days		–0.14 (–0.30 to 0.02)		0.02 (–0.09 to 0.13)		1.04
45 days		–0.17 (–0.36 to 0.02)		–0.08 (–0.21 to 0.05)		0.93
60 days		–0.21 (–0.43 to 0.01)		–0.03 (–0.18 to 0.12)		0.93
90 days		–0.27 (–0.52 to –0.02)*		–0.03 (–0.20 to 0.13)		0.83
180 days		–0.13 (–0.37 to 0.11)		–0.05 (–0.21 to 0.10)		0.59
CI, confidence interval. **a**Regression coefficient representing the change (%5mC per IQR in pollutant). *p ≤ 0.05.						

In multivariable models, we found that Alu methylation decreased with an IQR increase in ambient BC exposure (Table 2). The strongest associations for ambient BC exposure were during the periods 45–90 days before the examination. We also found evidence that LINE-1 methylation decreased with an IQR increase in BC exposure over prolonged periods, primarily during the periods 28–60 days before examination. An IQR increase in SO_4_ exposure during 90 days before examination was significantly associated with decreased LINE-1 methylation. We found no significant associations for PM_2.5_.

We conducted a sensitivity analysis by limiting our observations to those subjects who lived within a 40-km radius from the primary stationary air pollution monitor (*n* = 501). The point estimates obtained for this restricted analysis were similar (data not shown) to those observed with the complete data set; however, the standard errors increased. As a second sensitivity analysis, we modeled season through a sine and cosine function of time. In this analysis, we found nearly identical results for LINE-1. Our results for Alu shifted slightly, such that the longer averaging periods became more relevant. We found significant negative associations between BC and Alu at 90 and 180 days before examination. Similar to the main analysis, we found no significant associations between SO_4_ and Alu [see Supplemental Material, Table S.2 (doi:10.1289/ehp.1002773)].

**Figure 1:**
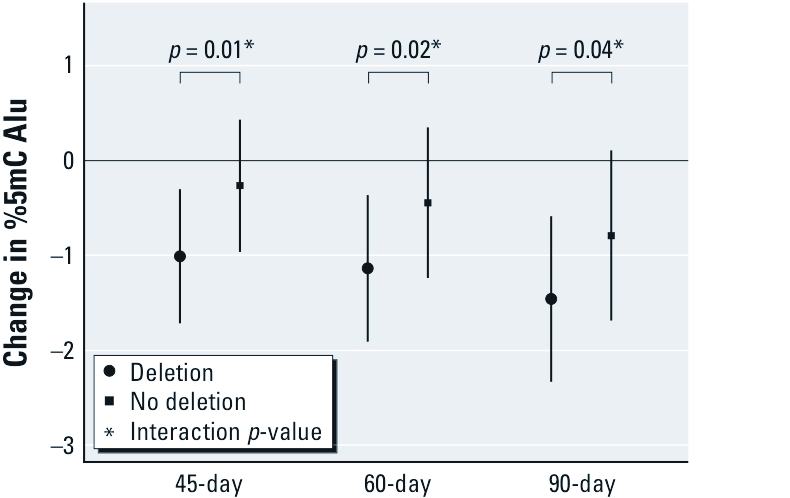
Association between BC and Alu according to time window of exposure and *GSTM1* genotype.

We focused our interaction analysis on the time periods where we saw most of the significant associations in our main effects analysis. We therefore examined interaction terms between BC and SO_4_ exposure during the periods 45, 60, and 90 days before the examination and genetic variants and host characteristics. We found that a deletion of *GSTM1* modified the association between BC exposure and Alu methylation. In all exposure periods, associations of BC with hypomethylation of Alu were stronger among those with the *GSTM1* null genotype than other participants (Figure 1). We found no significant interaction between BC and *GSTP1* or *GSTT1* genotypes; between SO_4_ and any genetic variant; for any of the genetic variants and either BC or SO_4_ on the change in LINE-1; or with any of the host characteristics evaluated as potential effect measure modifiers (i.e., current smoking, obesity, or use of antihypertensive medications) for either BC or SO_4_.

## Discussion

We found that increased exposure to BC over a prolonged period was associated with decreased methylation of Alu repetitive elements. The relevant time window of exposure to BC was between 45 and 90 days preceding the examination visit when DNA methylation was measured. We also found that increased exposure to BC over a period of 28–60 days before the examination was associated with decreased LINE-1 methylation. Increased exposure to SO_4_ over 90 days was associated with decreased methylation of LINE-1 repetitive elements.

The role of hypomethylation of retrotransposable elements in air pollution toxicity may be explained by their involvement in cellular stress and inflammation. Endogenous retroelements have been implicated in the regulation of cell stress responses and of the immune system and in the pathogenesis of several human autoimmune and inflammatory diseases. The strongest evidence linking human retroelement regulation by cell stress is on Alu sequences (Li and Schmid 2001). However, LINE-1 sequences might also be induced during cellular stress responses (Li and Schmid 2001), as well as in response to cytotoxic and proinflammatory stimuli such as cytotoxic chemotherapy (Hagan and Rudin 2002) and ultraviolet exposure (Banerjee et al. 2005). Furthermore, LINE-1 has been shown to be hypomethylated in fibroblasts from patients with rheumatoid arthritis, which are aberrantly activated and show enhanced proliferation, migration, and secretion of cytokines, chemokines, and proteases (Neidhart et al. 2000; Seemayer et al. 2001). Taken together, these data suggest that repetitive element hypomethylation participates in enhancing immune and inflammatory responses.

A very limited number of epidemiologic studies have looked at the association between PM and DNA methylation. Tarantini et al. (2009) examined genomic methylation content estimated by percent methylation within Alu and LINE-1 repeated elements in an occupational cohort exposed to PM_10_ on the first day of the work week and 3 days later. Although methylation did not show changes over the course of the work week, PM_10_ exposure levels were negatively associated with methylation in both Alu and LINE-1 when comparing measures of all subjects across various PM_10_ exposure levels, suggesting associations with long-term PM_10_ exposure. Breton et al. (2009) demonstrated that children exposed *in utero* to maternal smoking had significantly lower DNA methylation in Alu compared with those who were not exposed. Baccarelli et al. (2009) demonstrated evidence of an acute effect of BC on LINE-1 methylation in the same cohort that we have investigated here. Interestingly, we found that over prolonged periods of exposure, BC was associated with a decrease in Alu and LINE-1 methylation, whereas Baccarelli et al. (2009) saw an association with LINE-1 only when examining acute exposures (i.e., moving averages of ≤ 7 days). Additionally, we saw an association with SO_4_ and LINE-1 over a prolonged exposure period, although the prior study found no association with acute exposure to SO_4_ and either LINE-1 or Alu. In urban areas, BC derives primarily from exhaust emissions from vehicles (Janssen et al. 2002). In the U.S. Northeast, SO_4_ is an indicator of coal combustion PM. Our results indicate that both sources of this PM may induce epigenetic changes and suggest that they should remain a regulatory concern.

Our results suggest that prolonged exposures to BC and SO_4_ are important in hypomethylation of DNA repetitive elements. In time-series investigations of mortality, studies of PM_10_ exposure up to a month before death have shown greater associations than those with exposures a few days before death (Schwartz 2000; Zanobetti et al. 2002). Furthermore, cohort studies (Laden et al. 2006; Puett et al. 2009; Schwartz et al. 2008) have shown that the strongest associations between air pollution PM and cardiovascular morbidity and mortality are due to the exposures that take place within 1–2 years of the event. Such findings suggest that longer periods of cumulative exposure to PM air pollution may be important when examining mechanisms of toxicity. Although our results demonstrate that longer periods of cumulative exposure are associated with DNA hypomethylation, whether it is part of the pathway by which PM pollution affects cardiovascular morbidity and mortality cannot be determined by our observational design. An alternative explanation for our findings may be that decreased methylation simply reflects the up-regulation of secondary detoxification pathways as part of the normal response to PM exposure and that interindividual variation may lead some persons to be more susceptible than others.

Although the precise role of repetitive element hypomethylation in cardiovascular morbidity and mortality is unclear, a large and growing body of evidence indicates that transposable elements play a role in human development, gene expression, and human disease (Belancio et al. 2008, 2009; Han and Boeke 2005). Furthermore, small changes in methylation of repetitive elements may be clinically meaningful. The magnitude of hypomethylation that we found in the present study (Table 2) is of the same order of magnitude as that which has been associated with a 2-year increase in age (Bollati et al. 2009) and that corresponding to a 5% increased risk of developing ischemic heart disease (Baccarelli et al. 2010b) in this cohort.

Glutathione pathways are important in cellular defense against reactive oxygen species and have been shown to modify the effects of air pollution (Chahine et al. 2007; Schwartz et al. 2005). Depletion of glutathione has also been shown to alter DNA methylation (Lertratanangkoon et al. 1997). During prooxidant states, homocysteine is diverted away from the methionine cycle and toward the production of glutathione. This leads to a deficiency in methyl donors and genomewide DNA hypomethylation (Hitchler and Domann 2009). Our study demonstrates that the association between air pollution exposure and decreased DNA methylation may be enhanced in subjects with a *GSTM1* deletion. These findings agree with a study in 348 children, where prenatal exposure to tobacco smoke was associated with lower LINE-1 methylation in the *GSTM1*-null children but higher methylation in the *GSTM1*-present children (Breton et al. 2009). This is consistent with the hypothesis that individuals lacking the GSTM1 enzyme may compensate by increasing glutathione production, shunting cysteine from methionine synthesis and decreasing the availability of *S*-adenosyl methionine for DNA methylation. In addition, oxidative species can produce DNA damage that may reduce the capacity of methyltransferases to methylate the DNA and progressively induce DNA hypomethylation at each cell division (Baccarelli and Bollati 2009).

One major limitation of our study is that we were unable to measure the expression of LINE-1–encoded and Alu-encoded RNAs and proteins. Therefore, the potential roles of repetitive element hypomethylation in response to the exposure remain speculative. This study made use of a methylation assay that produces a cumulative measure of repetitive element sequences dispersed throughout the genome, regardless of their position (Yang et al. 2004). Although the assay we used is amenable for use in relatively large populations, such as the one we investigated, it does not provide data on locus-specific methylation. We cannot know if reduced methylation of LINE-1 was within the roughly 1% of LINE-1 sequences that can be transcribed. However, because of financial and sample quantity constraints, we felt that this assay was a good alternative to direct measurement of methyl group content through high-performance liquid chromatography. We also performed methylation analysis on white blood cell (WBC) DNA. To what extent the change we observed in WBC DNA reflects modification of DNA methylation in target tissues is unclear. However, WBCs regulate the systemic response to inflammation, and given that inflammation may be a relevant mechanism in the relationship between air pollution exposure and DNA methylation, measurements in WBCs may be biologically relevant.

Another limitation of this study is the use of a single ambient monitor to characterize exposure. The use of ambient concentrations as a surrogate for personal exposure can lead to exposure error when the pollutant is spatially heterogeneous. However, concentrations of PM_2.5_ have been shown to be homogeneous over a wide geographic region, and in panel studies in Boston (Koutrakis et al. 2005; Sarnat et al. 2005), where participants were longitudinally followed, longitudinal variation in ambient PM_2.5_ concentrations were strongly correlated with corresponding personal PM_2.5_ exposures. A more recent study specifically looked at long-term differences in ambient and personal levels of exposure to SO_4_, as a surrogate for PM_2.5_, averaged over time (Sarnat et al. 2009). Although differences between averaged ambient and personal SO_4_ were found within cities, the study still found mean ratios of personal to ambient exposure of 0.83 in Boston during the summer and approximately 0.6 during the winter.

With respect to the particular components of PM_2.5_, the spatial homogeneity may vary. SO_4_, a major component of PM_2.5_ in Boston, is also a stable particle species that varies little outdoors over wide geographic areas (Suh et al. 1995). In comparison, BC is moderately heterogeneous because of the numerous local sources (Park et al. 2006). Even so, although BC in a higher-traffic neighborhood is higher than in a lower-traffic neighborhood, the same processes (mixing height and wind speed) drive daily variation in both locations, and the longitudinal correlation is much better than the cross-sectional one. Nevertheless, we expect greater exposure error for this pollutant than for the others. Classical measurement error tends to bias the effect downward, whereas Berkson measurement error tends to increase the standard error of the estimate. When looking at longitudinal variations in air pollution, most error is of the Berkson type. To the extent that it is classical, simulation studies have shown that it is highly unlikely to bias away from the null even in the presence of covariates (Zeger et al. 2000). Therefore, measurement error in our BC exposure metric would likely attenuate the true association. Given that we found significant associations for BC, it is unlikely that this error would qualitatively change our conclusions.

As with any observational study, the possibility of residual confounding cannot be ruled out. However, because we investigated a time-varying exposure, a potential confounder would have to not only predict DNA methylation but also vary with time. We adjusted for several potential time-varying confounders (i.e., age, time trend, temperature, and season) and have included a random intercept for each subject in our mixed-effects models to minimize unmeasured, time-invariant confounding. We note that our method of adjustment for season, which has previously been shown to be associated with DNA methylation in this cohort (Baccarelli et al. 2009), may contribute to some model misspecification. We saw subtle changes in our results for Alu, depending on the method of seasonal adjustment, perhaps indicating that our model using indicator variables was “overadjusted.” We therefore urge caution in interpretation of our results. Rather than focusing on one particular exposure period, we emphasize that prolonged periods of exposure, ranging from 1 to 6 months, to PM air pollution are relevant in DNA hypomethylation.

The adverse effects of air pollution are greater in the elderly (Medina-Ramon and Schwartz 2008) and those with comorbid conditions, such as obesity (Dubowsky et al. 2006; Madrigano et al. 2010; Schwartz et al. 2005) and diabetes (Bateson and Schwartz 2004; O’Neill et al. 2007). Therefore, our findings may not be generalizable to other populations, because our cohort consists of elderly men, many of whom have comorbid conditions. Relevant findings will need to be replicated in other populations.

## Conclusion

Our results indicate that repetitive element methylation varied with prolonged exposure to ambient BC and SO_4_ among a population of elderly, community-dwelling men. In contrast to associations with acute exposure to particles, prolonged exposure to both SO_4_ and BC was associated with hypomethylation of two types of repetitive elements, Alu and LINE-1. This association was modified by a deletion of *GSTM1*. Whether these epigenetic changes contribute to or modify the effects of air pollution on cardiovascular morbidity and mortality merits further investigation.

## Supplemental Material

(188 KB) PDF
