# Supplementary material for: Prolonged Exposure to Particulate Pollution, Genes Associated with Glutathione Pathways, and DNA Methylation in a Cohort of Older Men
Source: Environ Health Perspect. 2011 Mar 8;119(7):977–82. doi: 10.1289/ehp.1002773 (PMC3222977; doi:10.1289/ehp.1002773)
Supplement: (188 KB) PDF [file ehp.1002773.s001.pdf]

Supplemental Material

Prolonged Exposure to Particulate Pollution, Genes Associated With Glutathione Pathways and DNA Methylation in a Cohort of Older Men

Jaime Madrigano
Andrea Baccarelli
Murray A. Mittleman
Robert O. Wright
David Sparrow
Pantel S. Vokonas
Letizia Tarantini
Joel Schwartz

Genotyping Methods

GSTM1

Genotyping for the glutathione *S*-transferase mu-1 (*GSTM1*) deletion copy number variant (UniGene Hs.301961; (UniGene 2010b)) was done at the Harvard School of Public Health. The assay consists of polymerase chain reaction (PCR) amplification of exons 4 and 5 of the *GSTM1* allele. Because this polymorphism is a gene deletion, the PCR product indicates the presence of at least one copy of the gene. Subjects who are homozygous null are therefore compared to subjects with 1 or 2 copies of the gene. In each case, concomitant amplification of the cytochrome P450 1A1 gene (*CYP1A1*) (UniGene Hs.72912; (UniGene 2010a)) was done as a positive control. The PCR amplification of *CYP1A1* results in a 312 base-pair product that is easily visualized in the presence or absence of the *GSTM1* 273 base-pair PCR product. The DNA template was prepared by mixing whole blood (0.5–1.0 μL) with reaction buffer and primers, followed by incubation. To this, 2.5 U Amplitaq was added; PCR was then performed, yielding the 273 base-pair product.

GSTP1

We genotyped two single nucleotide polymorphisms (SNPs) on the glutathione *S*-transferase pi-1 gene (*GSTP1*) (UniGene Hs.523836; (UniGene 2010c)): Ile105Val (A313G, rs1695), caused by an A-to-G substitution that changes codon 105 from ATC (Ile) to GTC (Val); and Ala114Val (C2293T, rs1799811), a C-to-T substitution that changes codon 114 from GCG (Ala) to GTG (Val) (Ali-Osman et al. 1997). Both SNPs were genotyped using unlabeled mini-sequencing reactions and mass spectrometry via a

Sequenom Mass Array MALDI-TOF (matrix-assisted laser desorption ionization time-of-flight) mass spectrometer (Sequenom, San Diego, CA) (Sun et al. 2000).

GSTT1

Analyses were performed using the TaqMan 5′ nuclease real-time quantitative PCR assay. Subjects were genotyped for glutathione *S*-transferase theta-1 gene (*GSTT1*) deletions (UniGene Hs.268573; (UniGene 2010d)). For this analysis, the following primers and probe were used: *GSTT1* exon 1 forward primer (5′-caggtcagcacttaagcgatgc-3′), *GSTT1* reverse primer (5′-cagcagtcctaggg tcccagtta-3′), and *GSTT1* probe (5′-Famccttcaaggctggcaccttcttgagg-Tamra-3′).

Table S.1. Distribution of pollutants by season and over entire study period, March 1999 – November 2007 (μg/m3)

Pollutant	Averaging Period	Season	Mean (SD)	IQR	ANOVA *P*-value[a]
$PM_{2.5}$	28-day	Spring	8.76 (1.73)	1.97	<0.0001
		Summer	12.60 (2.80)	3.01	
		Fall	9.39 (1.60)	2.25	
		Winter	10.66 (2.13)	3.36	
		All	10.31 (2.58)	3.33	
	45-day	Spring	8.83 (1.58)	2.03	<0.0001
		Summer	12.16 (2.71)	3.39	
		Fall	9.75 (1.79)	2.25	
		Winter	10.42 (1.84)	2.72	
		All	10.29 (2.39)	2.97	
	60-day	Spring	9.00 (1.54)	2.10	<0.0001
		Summer	11.61 (2.43)	2.92	
		Fall	10.19 (1.91)	2.21	
		Winter	10.26 (1.72)	2.61	
		All	10.29 (2.17)	2.66	
	90-day	Spring	9.43 (1.53)	2.12	<0.0001
		Summer	10.83 (1.97)	2.89	
		Fall	11.04 (1.94)	2.32	
		Winter	10.03 (1.51)	2.80	
		All	10.42 (1.91)	2.32	
	180-day	Spring	9.98 (1.24)	2.16	<0.0001
		Summer	10.20 (1.24)	1.52	
		Fall	10.98 (1.22)	2.10	
		Winter	10.62 (1.31)	1.64	
		All	10.47 (1.31)	1.53	

Table S.1 (continued)

Pollutant	Averaging Period	Season	Mean (SD)	IQR	ANOVA *P*-value[a]
BC	28-day	Spring	0.60 (0.13)	0.21	<0.0001
		Summer	0.86 (0.16)	0.21	
		Fall	0.83 (0.14)	0.21	
		Winter	0.76 (0.18)	0.30	
		All	0.77 (0.18)	0.27	
	45-day	Spring	0.61 (0.13)	0.18	<0.0001
		Summer	0.84 (0.16)	0.24	
		Fall	0.84 (0.13)	0.22	
		Winter	0.76 (0.16)	0.26	
		All	0.77 (0.17)	0.27	
	60-day	Spring	0.62 (0.14)	0.17	<0.0001
		Summer	0.80 (0.15)	0.22	
		Fall	0.86 (0.13)	0.21	
		Winter	0.76 (0.14)	0.25	
		All	0.77 (0.17)	0.25	
	90-day	Spring	0.65 (0.15)	0.18	<0.0001
		Summer	0.75 (0.14)	0.19	
		Fall	0.88 (0.12)	0.23	
		Winter	0.78 (0.12)	0.19	
		All	0.77 (0.16)	0.24	
	180-day	Spring	0.73 (0.14)	0.23	<0.0001
		Summer	0.72 (0.12)	0.19	
		Fall	0.82 (0.11)	0.18	
		Winter	0.84 (0.11)	0.20	
		All	0.77 (0.13)	0.21	

Table S.1 (continued)

Pollutant	Averaging Period	Season	Mean (SD)	IQR	ANOVA *P*-value[a]
SO_4	28-day	Spring	2.72 (0.51)	0.73	<0.0001
		Summer	3.92 (0.96)	1.40	
		Fall	2.82 (0.63)	0.96	
		Winter	2.69 (0.52)	0.56	
		All	3.04 (0.85)	1.04	
	45-day	Spring	2.72 (0.39)	0.53	<0.0001
		Summer	3.73 (0.78)	1.09	
		Fall	3.04 (0.69)	0.97	
		Winter	2.62 (0.42)	0.51	
		All	3.06 (0.75)	0.93	
	60-day	Spring	2.73 (0.36)	0.45	<0.0001
		Summer	3.56 (0.74)	0.90	
		Fall	3.20 (0.65)	0.88	
		Winter	2.59 (0.38)	0.49	
		All	3.06 (0.69)	0.93	
	90-day	Spring	2.76 (0.33)	0.48	<0.0001
		Summer	3.33 (0.57)	0.68	
		Fall	3.51 (0.58)	0.92	
		Winter	2.60 (0.30)	0.41	
		All	3.12 (0.61)	0.83	
	180-day	Spring	2.68 (0.23)	0.36	<0.0001
		Summer	3.04 (0.33)	0.43	
		Fall	3.43 (0.29)	0.40	
		Winter	3.12 (0.35)	0.52	
		All	3.10 (0.41)	0.59	

[a] ANOVA test comparing the pollutant mean across seasons.

Table S.2. Change in LINE-1 and Alu methylation (% 5mC) associated with an Interquartile Range (IQR) increase in Pollutant, Adjusting for season through a sine and cosine function of time

Exposure	LINE-1 beta[a] (95% CI)	Alu beta (95% CI)	IQR (μg/m3)
$PM_{2.5}$			
28-day avg	-0.03 (-0.17, 0.11)	0.03 (-0.06, 0.12)	3.33
45-day avg	-0.04 (-0.18, 0.10)	0.04 (-0.05, 0.13)	2.97
60-day avg	-0.05 (-0.19, 0.09)	0.05 (-0.04, 0.14)	2.66
90-day avg	-0.01 (-0.15, 0.14)	0.04 (-0.05, 0.14)	2.32
180-day avg	0.05 (-0.07, 0.17)	0.08 (0.00, 0.16)*	1.53
Black Carbon			
28-day avg	-0.22 (-0.43, -0.01)*	0.01 (-0.13, 0.15)	0.27
45-day avg	-0.31 (-0.58, -0.04)*	-0.13 (-0.31, 0.05)	0.27
60-day avg	-0.29 (-0.58, 0.00)*	-0.18 (-0.37, 0.00)	0.25
90-day avg	-0.35 (-0.68, -0.01)*	-0.42 (-0.64, -0.20)*	0.24
180-day avg	0.11 (-0.31, 0.52)	-0.29 (-0.56, -0.01)*	0.21
Sulfate			
28-day avg	-0.14 (-0.30, 0.03)	0.02 (-0.09, 0.13)	1.04
45-day avg	-0.15 (-0.35, 0.04)	-0.09 (-0.22, 0.04)	0.93
60-day avg	-0.19 (-0.42, 0.03)	-0.05 (-0.20, 0.10)	0.93
90-day avg	-0.30 (-0.56, -0.05)*	-0.05 (-0.23, 0.12)	0.83
180-day avg	-0.15 (-0.40, 0.10)	-0.01 (-0.17, 0.16)	0.59

[a] Regression coefficient representing the change (%5-methyl-cytosine / IQR in pollutant).
* $p \leq 0.05$

References

Ali-Osman F, Akande O, Antoun G, Mao JX, Buolamwini J. 1997. Molecular cloning, characterization, and expression in escherichia coli of full-length cDNAs of three human glutathione S-transferase pi gene variants. evidence for differential catalytic activity of the encoded proteins. J Biol Chem 272(15):10004-10012.

Sun X, Ding H, Hung K, Guo B. 2000. A new MALDI-TOF based mini-sequencing assay for genotyping of SNPS. Nucleic Acids Res 28(12):E68.

UniGene. 2010a. Cytochrome P450, Family 1, Subfamily A, Polypeptide 1. http://www.ncbi.nlm.nih.gov.ezp-prod1.hul.harvard.edu/UniGene/clust.cgi?UGID=139520&TAXID=9606&SEARCH=cyp1a1 ed. Bethesda, MD:National Center for Biotechnology Information.

UniGene. 2010b. Glutathione S-Transferase Mu 1. http://www.ncbi.nlm.nih.gov.ezp-prod1.hul.harvard.edu/UniGene/clust.cgi?UGID=181321&TAXID=9606&SEARCH=gstm1 ed. Bethesda, MD:National Center for Biotechnology Information.

UniGene. 2010c. Glutathione S-Transferase Pi 1. http://www.ncbi.nlm.nih.gov.ezp-prod1.hul.harvard.edu/UniGene/clust.cgi?UGID=914583&TAXID=9606&SEARCH=gstp1 ed. Bethesda, MD:National Center for Biotechnology Information.

UniGene. 2010d. Glutathione S-Transferase Theta 1. http://www.ncbi.nlm.nih.gov.ezp-prod1.hul.harvard.edu/UniGene/clust.cgi?UGID=172432&TAXID=9606&SEARCH=gstt1 ed. Bethesda, MD:National Center for Biotechnology Information.
